# Scene Configuration and Object Reliability Affect the Use of Allocentric Information for Memory-Guided Reaching

**DOI:** 10.3389/fnins.2017.00204

**Published:** 2017-04-13

**Authors:** Mathias Klinghammer, Gunnar Blohm, Katja Fiehler

**Affiliations:** ^1^Experimental Psychology, Justus-Liebig-UniversityGiessen, Germany; ^2^Centre for Neuroscience Studies, Queen's UniversityKingston, ON, Canada

**Keywords:** allocentric reference frames, scene coherence, memory-guided reaching, reliability, target-cue distance

## Abstract

Previous research has shown that egocentric and allocentric information is used for coding target locations for memory-guided reaching movements. Especially, task-relevance determines the use of objects as allocentric cues. Here, we investigated the influence of scene configuration and object reliability as a function of task-relevance on allocentric coding for memory-guided reaching. For that purpose, we presented participants images of a naturalistic breakfast scene with five objects on a table and six objects in the background. Six of these objects served as potential reach-targets (= task-relevant objects). Participants explored the scene and after a short delay, a test scene appeared with one of the task-relevant objects missing, indicating the location of the reach target. After the test scene vanished, participants performed a memory-guided reaching movement toward the target location. Besides removing one object from the test scene, we also shifted the remaining task-relevant and/or task-irrelevant objects left- or rightwards either coherently in the same direction or incoherently in opposite directions. By varying object coherence, we manipulated the reliability of task-relevant and task-irrelevant objects in the scene. In order to examine the influence of scene configuration (distributed vs. grouped arrangement of task-relevant objects) on allocentric coding, we compared the present data with our previously published data set (Klinghammer et al., 2015). We found that reaching errors systematically deviated in the direction of object shifts, but only when the objects were task-relevant and their reliability was high. However, this effect was substantially reduced when task-relevant objects were distributed across the scene leading to a larger target-cue distance compared to a grouped configuration. No deviations of reach endpoints were observed in conditions with shifts of only task-irrelevant objects or with low object reliability irrespective of task-relevancy. Moreover, when solely task-relevant objects were shifted incoherently, the variability of reaching endpoints increased compared to coherent shifts of task-relevant objects. Our results suggest that the use of allocentric information for coding targets for memory-guided reaching depends on the scene configuration, in particular the average distance of the reach target to task-relevant objects, and the reliability of task-relevant allocentric information.

## Introduction

We constantly interact with objects in our environment, like reaching for a mug or grasping a pen. In order to perform a goal-directed movement, the location of the object has to be encoded in the human brain which is then transformed into a motor plan and read-out by the motor system (Crawford et al., 2011). The brain makes use of multiple spatial reference frames to localize an object in space (Soechting and Flanders, 1992). Two broad classes of reference frames have been suggested, an egocentric and an allocentric reference frame (Colby, 1998; Klatzky, 1998; Battaglia-Mayer et al., 2003). In an egocentric reference frame, object locations are encoded relative to the observer, e.g., relative to the positions of the eyes, the head, or the body. It has been found that egocentric, and in particular gaze-centered, reference frames are predominantly used to encode targets for visually-guided reaching movements, but they are also involved in memory-guided movements (Lacquaniti and Caminiti, 1998; Cohen and Anderson, 2002; Fiehler et al., 2011; Thompson and Henriques, 2011). Besides egocentric coding schemes, allocentric reference frames also contribute to the encoding of movement targets (Diedrichsen et al., 2004; Krigolson and Heath, 2004; Obhi and Goodale, 2005; Krigolson et al., 2007; Byrne and Crawford, 2010). In an allocentric reference frame, object locations are encoded relative to other objects in the environment, background structures, or even imagined landmarks. There is evidence that allocentric coding is stronger for memory-guided than visually-guided reaching movements since they provide more stable, spatial information which can compensate for a rapid decline of visual target information (Bridgeman et al., 1997; Obhi and Goodale, 2005; Hay and Redon, 2006; Chen et al., 2011). However, allocentric coding schemes do also contribute to visually-guided reaching (Taghizadeh and Gail, 2014) supporting the notion of a combined use of egocentric and allocentric reference frames for visually-guided and memory-guided reaching movements.

Previous work on allocentric coding of reach targets mainly used simple and abstract stimuli, like LED light dots or bars, lacking ecological validity of the outcomes. In order to study allocentric coding of reach targets in more naturalistic scenarios, recent work from our lab applied naturalistic 2D images of complex scenes (Fiehler et al., 2014; Klinghammer et al., 2015) or 3D virtual reality (Klinghammer et al., 2016). In these experiments, we presented naturalistic images of a breakfast scene containing multiple objects on a table and in the background. Participants were instructed beforehand that either table or background objects function as potential reach targets and thus, are relevant for the task. Participants first encoded the scene with free gaze and after a short delay they briefly viewed a test scene with one of the task-relevant objects missing indicating the reach target location. After the test scene vanished, participants performed a reach to the remembered location of the missing object on a gray screen while gaze was fixed. Besides removing one object from the test scene, we also shifted some of the remaining objects either to the left or to the right. We found that reaching endpoints systematically deviated into the direction of object shifts, but only when task-relevant objects were shifted. When we shifted task-irrelevant objects that never became a reach target, reaching endpoints remained unchanged compared to a control condition with no object shifts. Because reaching endpoints were lying between the actual target location in the encoding scene and the target location relative to the shifted objects, we suggested that allocentric and egocentric information is integrated for memory-guided reaching. However, since we only varied the allocentric information by shifting the objects and kept most of the potential egocentric coding schemes constant (e.g., eyes, head or body), we cannot determine which egocentric information has been used in this task. One of our main findings was that only objects relevant for reaching served as potential landmarks and were used for allocentric encoding the target location. Thus, objects' task-relevance is an important factor determining whether they are used as allocentric cues or not. This is in line with previous findings showing that task relevance of objects in a scene leads to more and longer fixations on task-relevant objects (e.g., Ballard and Hayhoe, 2009) and can improve the detection of changes of object properties (Triesch et al., 2003) and their retention in visual working memory (Maxcey-Richard and Hollingworth, 2013).

**Figure 1:**
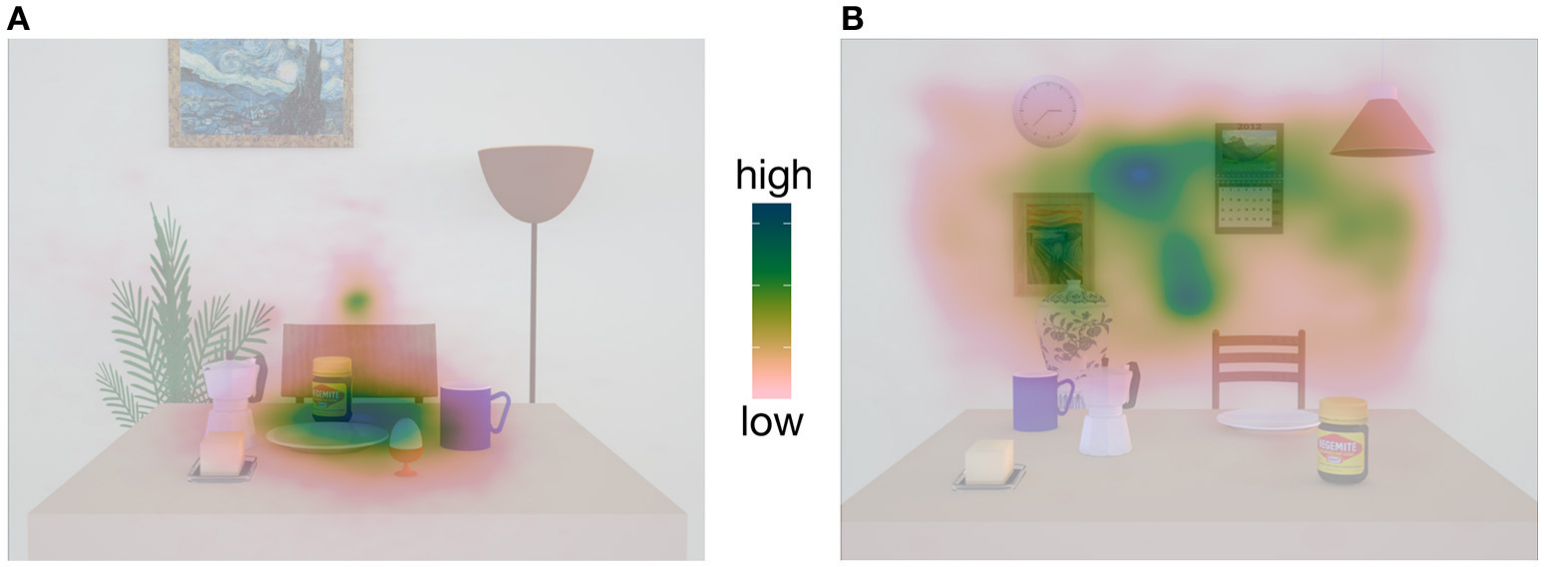
**Fixation density maps adapted from Klinghammer et al. (**2015**) containing examples images of the stimuli**. In **(A)**, only objects on the table served as reach targets (task-relevant objects) and formed a spatial object cluster which is spatially distinct from the irrelevant objects in the background. As a result, participants mainly fixated this area while ignoring the background. In **(B)**, only objects in the background served as reach targets and formed an object cluster. Consequently, we observed the reversed fixation pattern.

In our experiments so far (Fiehler et al., 2014; Klinghammer et al., 2015), task-relevant objects were located either on the table or in the background forming a spatial cluster that was separated from the cluster containing task-irrelevant objects (see Figure 1). As indicated by the fixation behavior, this spatial arrangement influenced participants' encoding strategies in a way that their overt spatial attention was mainly directed to the relevant object cluster while ignoring the area containing the irrelevant objects (see fixation density maps, Figure 1). The question arises whether spatial grouping of task-relevant information facilitates allocentric coding and thus, would be impeded if task-relevant objects are distributed across the whole scene. Moreover, spatial grouping of objects in task-relevant table or background objects also led to a smaller mean distance between the reach target and the task-relevant than task-irrelevant objects. There is evidence that with an increasing distance between target and landmark (i.e., allocentric cue), the landmark becomes less effective (Krigolson et al., 2007; Camors et al., 2015). Furthermore, endpoints of pointing movements are most affected by the closest landmark if multiple landmarks are available (Spetch, 1995; Diedrichsen et al., 2004). In this study, we aimed to examine the influence of the scene configuration by randomly placing task-relevant objects on the table *and* in the background within the same scene. By doing so, we not only increased the mean distance from the target to the task-relevant objects but at the same time also reduced the mean distance from the target to the task-irrelevant objects which also occurred in close proximity to the target comparable to the task-relevant ones. Based on the findings reported above, we predict a decreased contribution of allocentric information for spatial coding of reach targets compared to our previous study (Klinghammer et al., 2015), in which task-relevant objects were spatially grouped and therefore, placed in closer vicinity to the target than task-irrelevant objects.

Beyond the scene configuration, the reliability of allocentric cues might be an important factor determining their use for coding reach targets in space. Byrne and Crawford (2010) investigated the influence of the reliability and stability of landmarks on allocentric coding of target locations for memory-guided reaching. Landmarks consisted of four dots that were arranged in an imagined square around a target dot. They varied their stability and reliability by manipulating the amplitude of the dots' vibration. The authors hypothesized that with increasing the vibration amplitude the landmark stability and reliability should decrease and thus, the weighting of the allocentric information, should also decrease. As expected, they found a larger influence of low vibrating landmarks on direction-independent reaching endpoints compared to landmarks with high vibration amplitude; however, there was no effect on the variability of reaching endpoints. The authors concluded that landmark stability influences the weighting of the allocentric information. This is in line with previous studies on memory-guided reaching showing that humans preferably use stable and reliable landmarks leading to increased reaching endpoint accuracy (Krigolson and Heath, 2004; Obhi and Goodale, 2005). Similar results have been reported for changes in spatial object configurations influencing the reliability of allocentric cues. For example, when asking participants to detect a shift of one of multiple objects from an encoding to a probe display they were more accurate in conditions in which the objects were shifted coherently in one direction (minimal change) than they were arranged in a new, random fashion (maximal change) (Jiang et al., 2000). This suggests that the global configuration of objects in the display was taken into account and used for representing the single objects' location. The second goal of this study was to examine whether and how the reliability of task-relevant and task-irrelevant objects, i.e., the reliability of allocentric information, affects the use of objects as allocentric cues. We define reliability of allocentric information as the stability of objects in a scene, i.e., whether they are shifted coherently (stable/reliable) or incoherently (unstable/unreliable). We expect that breaking up the coherence of the spatial object configuration due to incoherent object shifts, the reliability of these objects and thus, their contribution to allocentric coding of reach targets should decrease.

In the current study, we used naturalistic images, similar to the ones published by Klinghammer et al. (2015), to study (1) the role of the scene configuration and (2) the object reliability on allocentric coding of target locations for memory-guided reaching. For answering the first question, we distributed task-relevant and task-irrelevant objects across the whole scene preventing spatially distinct clusters. Based on our previous findings (Klinghammer et al., 2015), we expected systematic deviations of reaching endpoints in the direction of task-relevant but not of task-irrelevant object shifts. However, reach endpoint deviations resulting from task-relevant object shifts should be smaller than the ones we observed by Klinghammer et al. (2015) due to the increased distance between the target and the task-relevant objects and/or the placement of task-irrelevant objects in closer proximity to the potential reach targets. In order to answer the second question, we manipulated the reliability of task-relevant and task-irrelevant objects by introducing conditions where we either kept the coherence of the whole object arrangement intact (coherent object shift direction) or broke it up (incoherent object shift direction). We expected that systematic deviations of reaching endpoints in the direction of object shifts are larger in the condition with high (intact coherence) than low (broken coherence) object reliability. Assuming that task-irrelevant objects are widely ignored and thus, unused for allocentric coding of memorized reach targets (Klinghammer et al., 2015), reach endpoint deviations in the direction of object shifts should be strongly influenced by the reliability of task-relevant objects, but hardly affected by the reliability of task-irrelevant objects. In particular, we expected an effect of object shifts on reaching endpoints in conditions in which we kept the scene coherence within the group of task-relevant objects intact, which should strongly decrease when we shifted task-relevant objects incoherently. In contrast, we expected a substantially reduced influence of object shifts in conditions when task-irrelevant compared to task-relevant objects are shifted alone regardless of the coherence manipulation.

## Materials and methods

### Participants

We recorded data from 22 participants. Four of them had more than 30% of trials without correct fixation and thus, were excluded from further analysis. For one additional participant, we failed to measure reach endpoints or correct fixation behavior in more than 60% of trials and therefore discarded the data from further analysis. The final sample consisted of 17 participants (8 female) with normal or corrected to normal vision ranging in age from 19 to 30 years (*mean* 25 ± *SD* 3.2 years). They were right-handed as assessed by Edinburgh handedness inventory (EHI, Oldfield, 1971; *mean handedness quotient* 78 ± *SD* 18.8). They received course credit or were paid for their participation. The experiment was approved by and conducted in agreement with the ethical guidelines of the local ethics committee of the University of Giessen in compliance with the Declaration of Helsinki (2008)^1^. Each participant signed the ethics form before the start of the experiment.

### Apparatus

Stimuli were presented on a 19″ (40.5 × 30 cm) CRT monitor (Ilyama Vision Master Pro 510) with a resolution of 1280 × 960 pixels and a refresh rate of 85 Hz. To reduce the influence of a high-contrast frame around the scene, a black cardboard (70 × 50 cm) frame was attached to the monitor. Participants sat at a table with their head stabilized on a chin rest with a distance of roughly 47 cm from the eyes to the center of the screen. A decimal-keyboard was placed in front of the subjects with the start button 24 cm away from the screen and aligned to the chin rest and the center of the screen. Reaches were performed with the right index finger and recorded with an Optotrak Certus (NDI, Waterloo, Ontario, Canada) tracking system with a sampling rate of 150 Hz using one infrared marker attached to the fingertip of the right index finger. To control for correct fixation behavior, eye movements were recorded using an EyeLink II system (SR Research, Osgoode, Ontario, Canada) with a sampling rate of 500 Hz. To run the experiment and to control the devices we used Presentation 16.5 (Neurobehavioral Systems, Inc., Berkeley, CA).

### Materials

Stimuli consisted of 3D-rendered images of a breakfast scene. Images were created in SketchUp Make 2015 (Trimble Navigation Ltd., Sunnyvale, CA) and afterwards rendered with Indigo Renderer 3.8.21 (Glare Technologies Ltd.) with a resolution of 3562 × 2671 pixels. The breakfast scene contained 5 objects consisting of a coffee mug, a plate, an espresso cooker, a Vegemite jar, and a butter dish placed on a brownish table that stood 86 cm in front of a gray wall. Furthermore, 6 objects, consisting of a chair, vase, painting, calendar, clock, and ceiling lamp were located behind the table in the background. Objects were taken from the open access online 3D-gallery of SketchUp. Object properties are summarized in Table 1.

**Table 1:** **Maximum height, width and distance to camera of all objects in the scene in cm, based on the actual properties in SketchUp**.

**Object**	**Height (visible)**	**Width**	**Distance to camera**
Plate	1.97	19.32	variable
Butter dish	4.89	8.36	variable
Espresso cooker	15.11	8.62	variable
Vegemite jar	11.44	6.86	variable
Mug	9.80	7.80	variable
Chair	18.00	18.00	229.32
Vase	31.98	19.69	241.52
Painting	29.11	22.59	variable
Calendar	31.21	19.28	variable
Clock	20.45	20.45	variable
Ceiling lamp	12.48	20.18	182.13

*Objects on the table, painting, calendar and clock had no fixed distance to the camera because they were randomly placed on one of three different depth lines on the table or their position altered on three different height levels at the wall, respectively. Some background objects were sometimes not fully visible due to object overlap. Therefore, visible heights may vary from the actual height depending on the object arrangement*.

We set all objects in 18 different arrangements (*encoding image*). They were placed so that <20% of an object was occluded by another object and with a distance to the edges of the table or the image so that in case of object displacement no object stood in the air next to the table or outside of the image. In any arrangement, objects on the table were placed at one of three possible horizontal depth lines that were equally spaced (19.5 cm starting from the front table edge) on the table with minimal 1 and maximal 2 objects positioned at every depth line. The painting, calendar and clock were placed at three different heights at the wall with 1 object placed at every height level, and the calendar never placed on the highest level in order to minimize unrealistic object arrangements in the scene. The distance of the low height from the ground was 107.55 cm, of the middle height 126.38 cm and of the high height 145.20 cm. Distances from the height levels to the camera were 278.97, 279.51, and 281.30 cm for the low, middle, and high height, respectively. The positions of the vase, chair, and ceiling lamp were fixed on one horizontal line for each object in different distances to the camera (see Table 1). Based on the encoding images, we created *test images*, in which 1 of 6 pre-defined objects (3 table objects and 3 background objects) was missing (= *reach target*). These 6 pre-defined objects served as potential reach targets and thus, were highly relevant to perform the task [ = *relevant objects* (RO)]. The remaining 5 objects never served as reach targets and thus, were task-irrelevant [ = *irrelevant objects* (IO)]. In 2/3 of the test images, objects (RO and/or IO) were shifted horizontally between 3.56 and 4.47° (*mean* 3.86° ± *SD* 0.33°) either to the left or to the right (50% leftward displacement) in the same (= coherent object shift) or in opposite directions (= incoherent object shift). Variations in the horizontal object displacement arose from the fact that objects were placed at different depth lines relative to the virtual camera position. Hence, similar physical shifts of objects at different depth lines in 3D-space would result in different displacements in the 2D-image. In the remaining 1/3 of the test images, no objects were shifted. These images served as control condition.

In total, 360 images were rendered, including 18 encoding images, 228 test images (76 with only RO shifts, 76 with only IO shifts, 76 with RO and IO shifts) and 114 control images. Moreover, from each of the 18 encoding images, a scrambled version made up of 768 randomly arranged squares of the image was created and used to mask the encoding image.

### Procedure

Participants first read a written instruction about the experimental procedure informing about the RO and their function. Afterwards, they performed a learning block in which the 6 RO were presented together on the computer screen and participants were requested to memorize these objects without time restriction. Next, a picture of only one RO or IO was presented and participants were asked to indicate by button press whether this object was a potential reach target or not. After feedback about the correctness of the response was given, the next picture with a different object appeared on the screen. This was repeated until every object was presented once. The learning block ended if participants correctly classified the presented objects as potential reach target for at least three times in a row. Then, the experiment started after some training trials.

**Figure 2:**
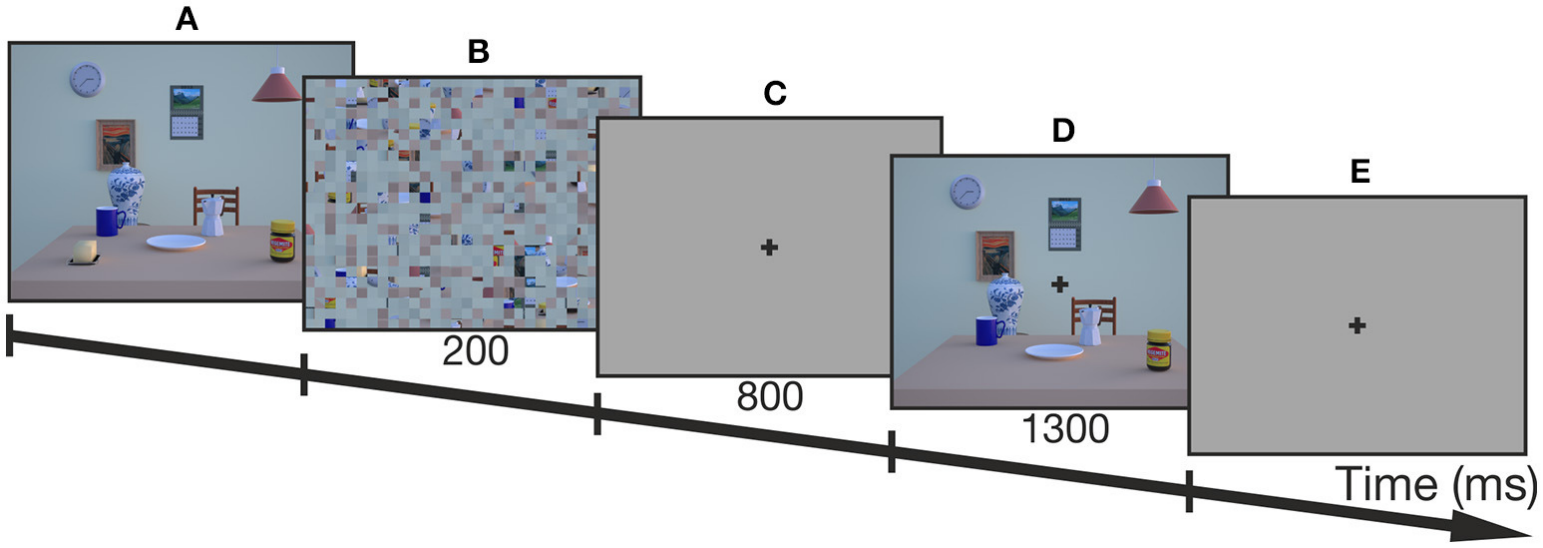
**Trial scheme of one example trial (control condition). (A)** First, the encoding image was presented and participants terminated the exploration of the image by button press. **(B)** Then, a scrambled version of the encoding image was presented for 200 ms, followed by **(C)** a delay which lasted for 800 ms. **(D)** Thereafter, the test image with one of the task-relevant objects missing (butter dish) was presented for 1300 ms before **(E)** a tone prompted participants to reach to the target onto a gray screen while fixating the cross at the center of the screen.

The procedure of an example trial is depicted in Figure 2. Before every trial, a fixation cross on a gray screen appeared prompting participants to fixate and press a button in order to perform a drift correction for the EyeLink II. Thereafter, the trial started with the presentation of the encoding image of the breakfast scene. Participants freely explored the scene without any time constraints and terminated the encoding phase by pressing the start button. Then, a scrambled version of the encoding scene appeared for 200 ms to avoid afterimages followed by a delay phase of 800 ms with a gray screen and a central fixation cross. Participants were instructed to fixate the cross until the end of the reaching movement in order to control for changes in gaze-centered, egocentric coding due to eye-movements (Thompson and Henriques, 2011). After the delay, the test image was presented for 1300 ms which lacked 1 RO defining the reach target. The trial continued with a short tone after the test image vanished which signaled the participants to perform the reaching movement toward the remembered location of the target object onto a gray screen. Thus, reaches were performed with gaze kept on the fixation cross and without any visual information of the encoding or test images. In this way we ensured that allocentric information could not be used for subsequent online corrections during the reaching movement, which would have led to an allocentric bias.

Participants were instructed to reach to the location of the missing object as accurately and natural as possible. Whenever they were unsure about the target location or identity, they had to reach to a marked location at the lower right edge of the monitor. These invalid trials were repeated at the end of the experiment. If subjects released the button before the go-signal, they received feedback and these invalid trials were also repeated at the end of the experiment.

**Figure 3:**
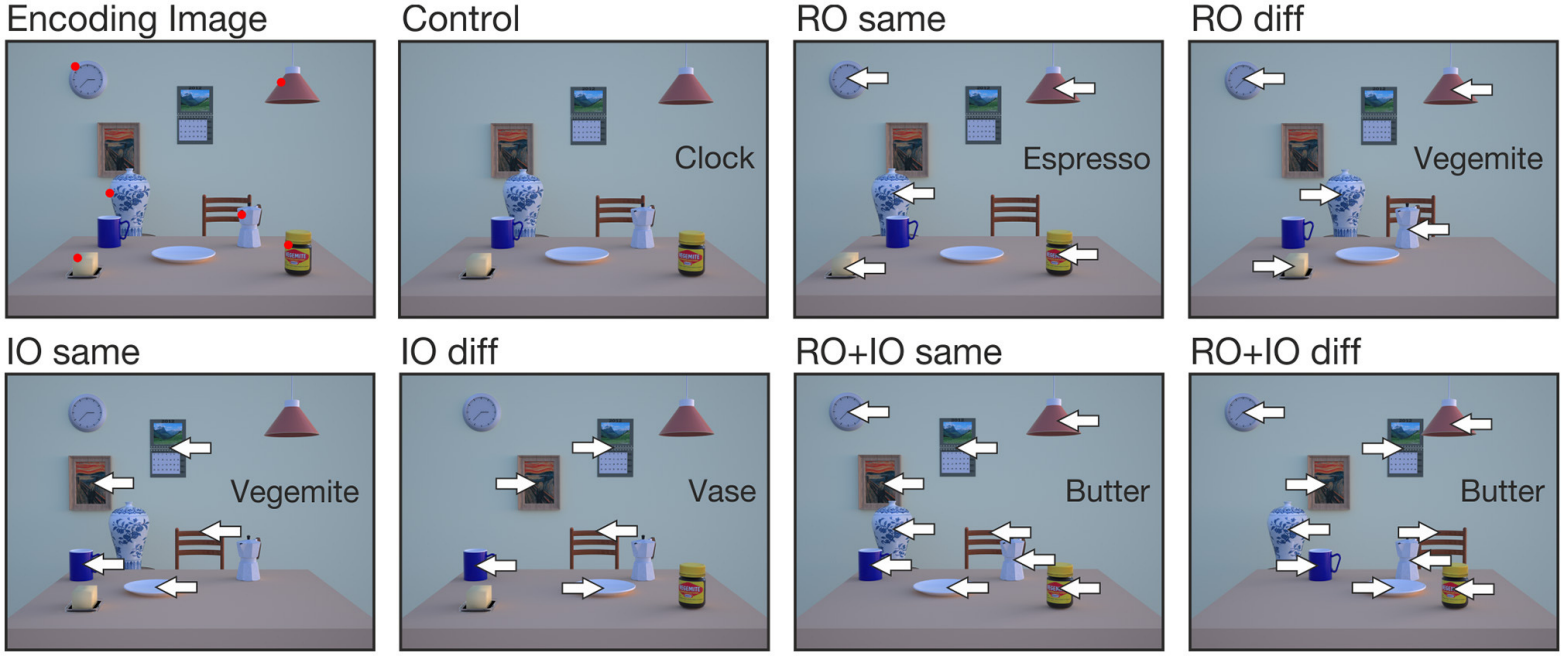
**Examples of encoding and test images for the 7 different conditions**. Object names in the box indicate the reach target (= missing object on the table). Arrows indicate the direction in which objects were shifted. Red dots in the encoding image mark the task-relevant objects (RO), but were absent in the experiment.

Participants performed six experimental conditions (for examples, see Figure 3). In all experimental conditions, 1 of 6 RO was always removed from the test image, which served as the reach target. In the *RO same* condition, the remaining 5 RO were shifted either to the left or to the right. In the *IO same* condition, all 5 IO were shifted left- or rightward. In the *RO diff* or *IO diff* condition, the 5 relevant or the 5 irrelevant objects were shifted in different directions with 3 objects displaced in one and the remaining 2 objects in the opposite direction, i.e., 3 objects shifted rightward and 2 leftward or vice versa. The direction in which 3 objects were shifted is regarded as the main shift direction. In the *RO*+*IO same* condition, all relevant and irrelevant objects were shifted in the same direction, whereas in the *RO*+*IO diff* condition all relevant objects were shifted in the opposite direction of all irrelevant objects. How these different conditions influence the overall scene coherence and the coherence within the group of task-relevant objects is summarized in Table 2. In all conditions, left- and rightward object shifts were balanced with 50% of trials in each direction; the same accounts for the direction of the main object shift in the conditions RO diff and IO diff. In the *control* condition, all objects remained stationary.

**Table 2:** **Expected influences of the different object shifts in the 7 conditions on the global scene coherence and the coherence of object locations within the group of RO and IO**.

**Condition**	**Influence on…**		
	**…Global coherence**	**…Coherence within RO**	**…Coherence within IO**
	
control	intact	intact	intact
RO same	partially broken	intact	intact
IO same	partially broken	intact	intact
RO diff	partially broken	broken	intact
IO diff	partially broken	intact	broken
RO+IO same	intact	intact	intact
RO+IO diff	broken	intact	intact

Each participant completed a minimum of 648 trials. Because some trials were repeated (criteria see above), the actual number of performed trials varied from 651 to 736 trials. Trials were separated in three sessions with one session per day which lasted about 1 h with one break in between. At the start of each session and after each break, the EyeLink II system was recalibrated. Trials were presented in pseudo-randomized order with a random sequence of conditions and encoding images within a session but fixed trial combinations between sessions. A trial was never followed by a trial containing the same encoding image. Every trial was repeated one time.

### Data reduction and statistical analysis

Data preprocessing was performed with MATLAB R2012a (The MathWorks Inc., Natick, MA) and inferential statistics with R 3.1 (R Development Core Team, http://www.r-project.org). All statistical tests were computed with an alpha-level of 0.05 after testing for normality (Kolmogorov-Smirnov test) and similarity of variances (Bartlett's test for planned *t*-tests, Mauchly's sphericity test for planned ANOVAs). If correction for multiple testing was necessary, Bonferroni-Holm correction was applied by multiplying the sorted *p*-values with the number of remaining tests. In case variances between samples were not equal, we used Welch approximation to the degrees of freedom for the *t*-tests and Greenhouse-Geisser correction for the ANOVAs. Effect sizes were calculated by using Hedges g for *t*-tests and the generalized Eta-Squared for ANOVA in case of significant results.

As a first step, we tested whether the group of relevant objects is different from the group of irrelevant objects regarding their saliency, which could have affected participant's attention, and thus, their encoding and reaching behavior. Therefore, we determined the mean saliency for every object in every encoding image by calculating the mean Graph-Based Visual Saliency (gbvs; Harel et al., 2007; Schuetz et al., 2016) of these objects. Next, we split these saliencies into two groups of relevant and irrelevant objects and averaged the mean saliencies for every object. After this, we entered the mean saliencies of these groups into a two-sided *t*-test.

Then, we inspected the eye tracking data and discarded trials from further data analysis in which subjects' gaze deviated more than 2.5° from the center of the fixation cross during a period beginning from delay onset till the end of the reaching movement. All in all 724 trials (6.57%) were rejected due to bad fixation. Second, reaching onsets and offsets were defined for every trial. The moment participants released the response button determined the reaching onset. Reach offsets were calculated from Optotrak data and defined as the first time point during the movement when velocity dropped below 20 mm/s if the index finger reached a maximum distance of about 3 cm from the screen. Reach endpoints were extracted at the time of reach offset. Some trials were excluded because reaching offsets or endpoints could not be extracted due to rarely occurring interferences of the infrared markers of the Optotrak with the IREDs of the EyeLink II (134 trials = 1.3%). Third, we excluded trials in which reaching endpoints deviated more than ± 2.5 *SD* in horizontal or vertical direction from the group mean in each condition for each object shift direction (534 trials = 5.26%). Taken together, from originally 11.016 trials of all participants, 9.624 valid trials (87.36%) remained.

To investigate the influence of object shifts (i.e., allocentric information) on reaching endpoints, we calculated allocentric weights for every subject and every condition by linear regression fit with an intercept set to zero. First, we determined reaching errors as the horizontal distance of the reach endpoint and the actual target position of the encoding image. Therefore, we averaged reach endpoints of the control condition of all subjects for every combination of object arrangements and target objects separately. Since none of the remaining objects were displaced in the control condition, we assume no systematic reaching errors. These averaged reach endpoints were used to define the target positions. Then, we calculated the differences of the reaching endpoints of the other experimental conditions from the corresponding target position in the horizontal plane. This resulted in positive values for reaching errors to the right and negative values for reaching errors to the left. In the next step, we determined *maximal expected reaching errors* (MERE) for every image after an object shift by assuming that participants completely relied on the allocentric information of the shifted objects (i.e., the information about the target location that is given by the surrounding non-target objects that were shifted) and thus produced reaching endpoints equal to the amount of the objects' displacement. To this end, we averaged the amount of displacement of the shifted objects for every image. If objects were shifted in different directions, we either averaged over the shift distances of only relevant objects (RO+IO diff) or averaged over the main shift direction (RO diff, IO diff). For the regression fit with a fixed intercept at zero, the MERE was used as a predictor and the actual reaching error as a dependent variable for the two shift directions within one condition for every subject. The resulting slope of the regression line indicated the extent to which a participant relied on the allocentric information of object displacements and thus was defined as allocentric weight. Therefore, an allocentric weight of 0 would indicate no use of the allocentric information of the shifted objects (equal to the no-shift control condition), whereas an allocentric weight of 1 would indicate a full use of the allocentric information of the shifted objects. These weights were determined across the two shift directions separately for every participant and every condition and then entered into further analyses. To investigate the influence of object shifts on the variability of reaching endpoints, we calculated standard deviations of reaching errors (in cm) for every participant in every condition. To account for the fact that reaching errors to the left had negative and reaching errors to the right had positive values, we calculated standard deviations for the two shift directions separately and averaged the data afterwards.

To investigate whether participants used a different encoding behavior with respect to their focus of overt visual attention compared to our previous experiments (Klinghammer et al., 2015), we created fixation density maps of participants' fixation behavior during the encoding phase. To this end, we calculated a mean fixation point for every fixation starting from the second fixation until the end of the encoding phase. To examine whether participants fixated relevant objects more often than irrelevant objects, we collapsed fixations of the different object arrangement scenes resulting in 18 different heatmaps. We then visually inspected the heatmaps and descriptively compared them to the ones of our previous study (Klinghammer et al., 2015).

Next, we performed two-sided one-sample *t*-tests to investigate whether the group allocentric weights of the different conditions differed significantly from zero. To investigate whether the scene configuration influenced the use of the allocentric information for memory guided reaching, we performed two-sided *t*-tests for independent samples on allocentric weights from the RO same condition of the current study and the corresponding conditions (conditions when five task-relevant objects were shifted in the same direction) of our previous study published by Klinghammer et al. (2015). In order to assess the impact of scene coherence and thus, the reliability of allocentric cues on reaching endpoints and their variability, we first conducted two-sided one-sample *t*-tests for the allocentric weights and standard deviations of reaching errors for the conditions RO+IO same and RO+IO diff. Second, we performed a two-way repeated measures ANOVA for conditions RO same, RO diff, IO same, and IO diff with the two factors shift coherence (same or different shift direction) and object relevance (shifted objects are potential reach targets or not). In case of significant main effects or interactions, we conducted two-sided *post-hoc t*-tests for paired samples.

## Results

First, we tested for differences in the saliency between task-relevant and task-irrelevant objects. Therefore, we determined the mean gbvs for every object of all encoding images and compared these mean saliency values between task-relevant and task-irrelevant objects. We found normally distributed (K-S test: *ps* > 0.765) mean saliency values that did not differ between task-relevant and task-irrelevant objects [*t*_(9)_ = −0.890, *p* = 0.397]. Thus, it is unlikely that participant's encoding and reaching behavior was affected by different object saliencies between these two groups.

**Figure 4:**
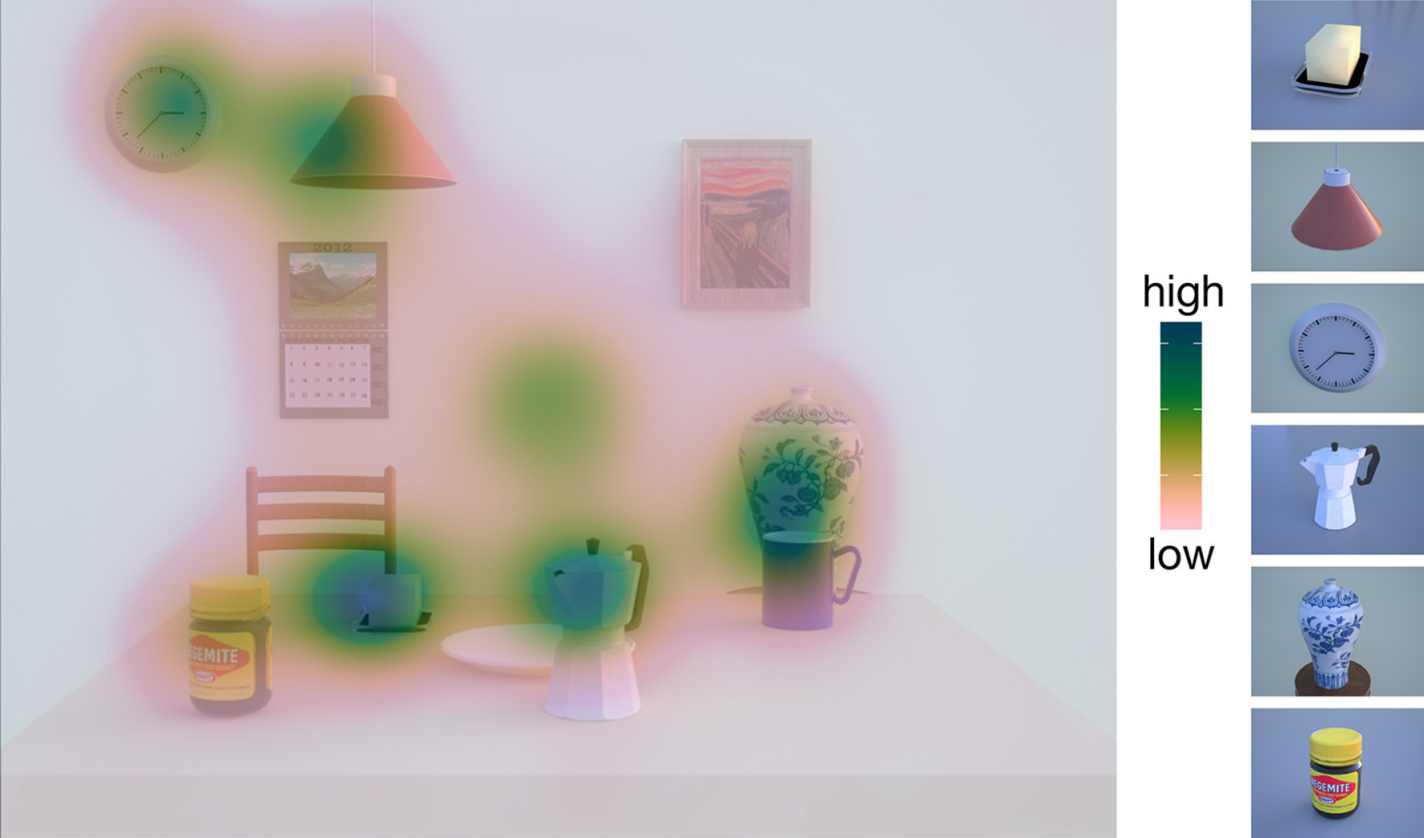
**Fixation density map with averaged fixations of all participants during the encoding phase for one example object arrangement**. On the right side, the task-relevant objects of the experiment are depicted (butter dish, ceiling lamp, clock, espresso cooker, vase, Vegemite jar). Participants show higher fixation frequencies for these task-relevant objects than for the remaining task-irrelevant objects.

To investigate the participants‘encoding behavior, we created fixation density maps of the encoding scene for every object arrangement. As an example, we depict one representative heatmap for one exemplary object arrangement in Figure 4. Fixation density was highest at the locations of task-relevant objects (butter dish, ceiling lamp, clock, espresso cooker, vase, Vegemite jar) whereas it was lower at locations of task-irrelevant objects. The heatmaps of the other object arrangements showed a very similar pattern.

**Figure 5:**
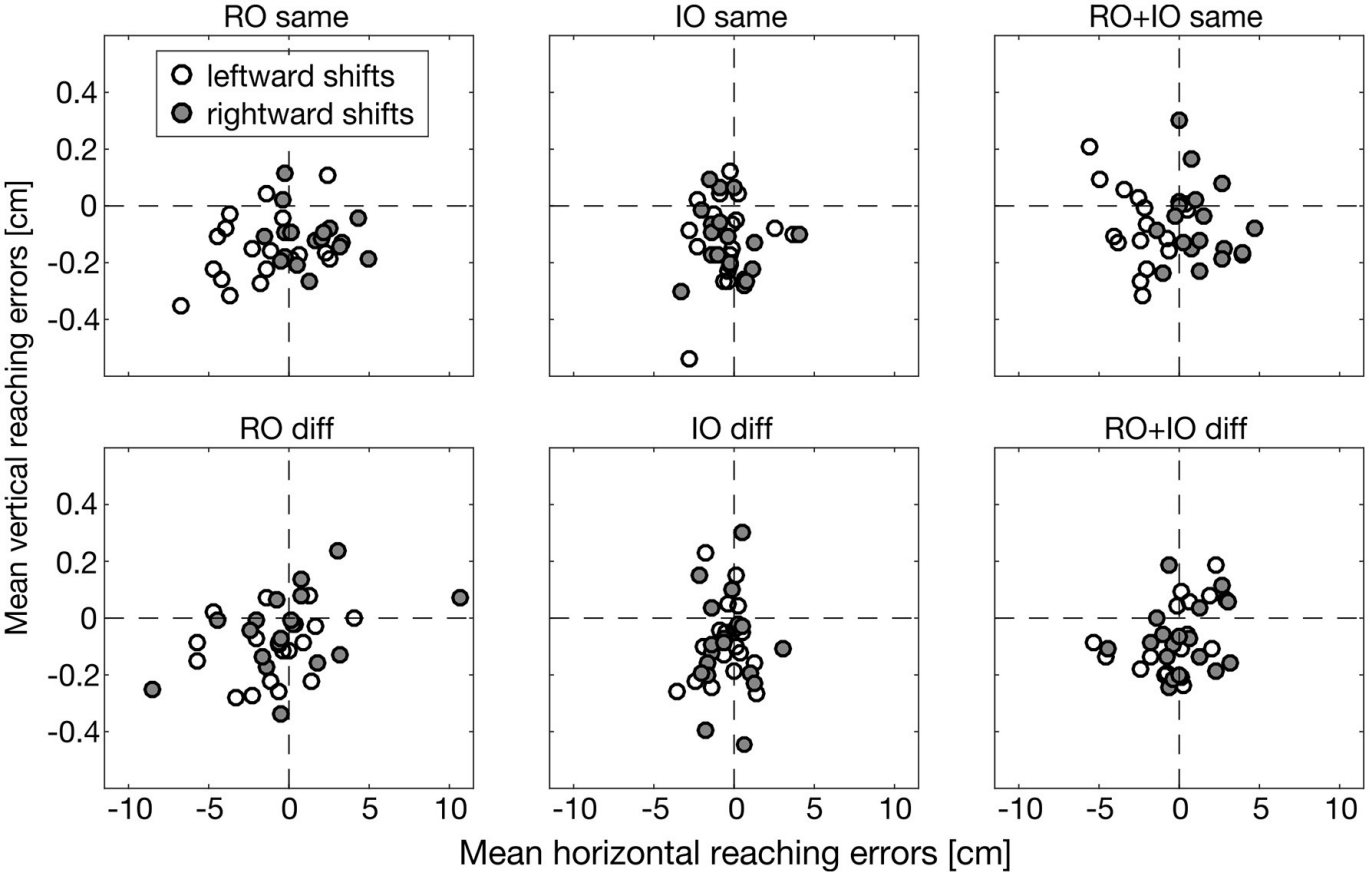
**Horizontal and vertical reaching errors (in cm) of one exemplary participant averaged across the different object arrangements**. Leftward object shifts are represented by white, rightward objects shifts by dark gray dots. Please note the small values on the ordinate.

In Figure 5, we illustrate reaching errors of one exemplary participant in the different conditions averaged over the 18 object arrangements. Reaching endpoints in conditions RO same and RO+IO same deviated systematically in the direction of object shifts. RO diff and RO+IO diff also showed horizontal reaching errors, but these errors were independent of the direction of object shifts. The conditions IO same and IO diff hardly demonstrated deviations of reaching endpoints indicating that in these conditions object shifts had a negligibly influence on reaching behavior.

**Figure 6:**
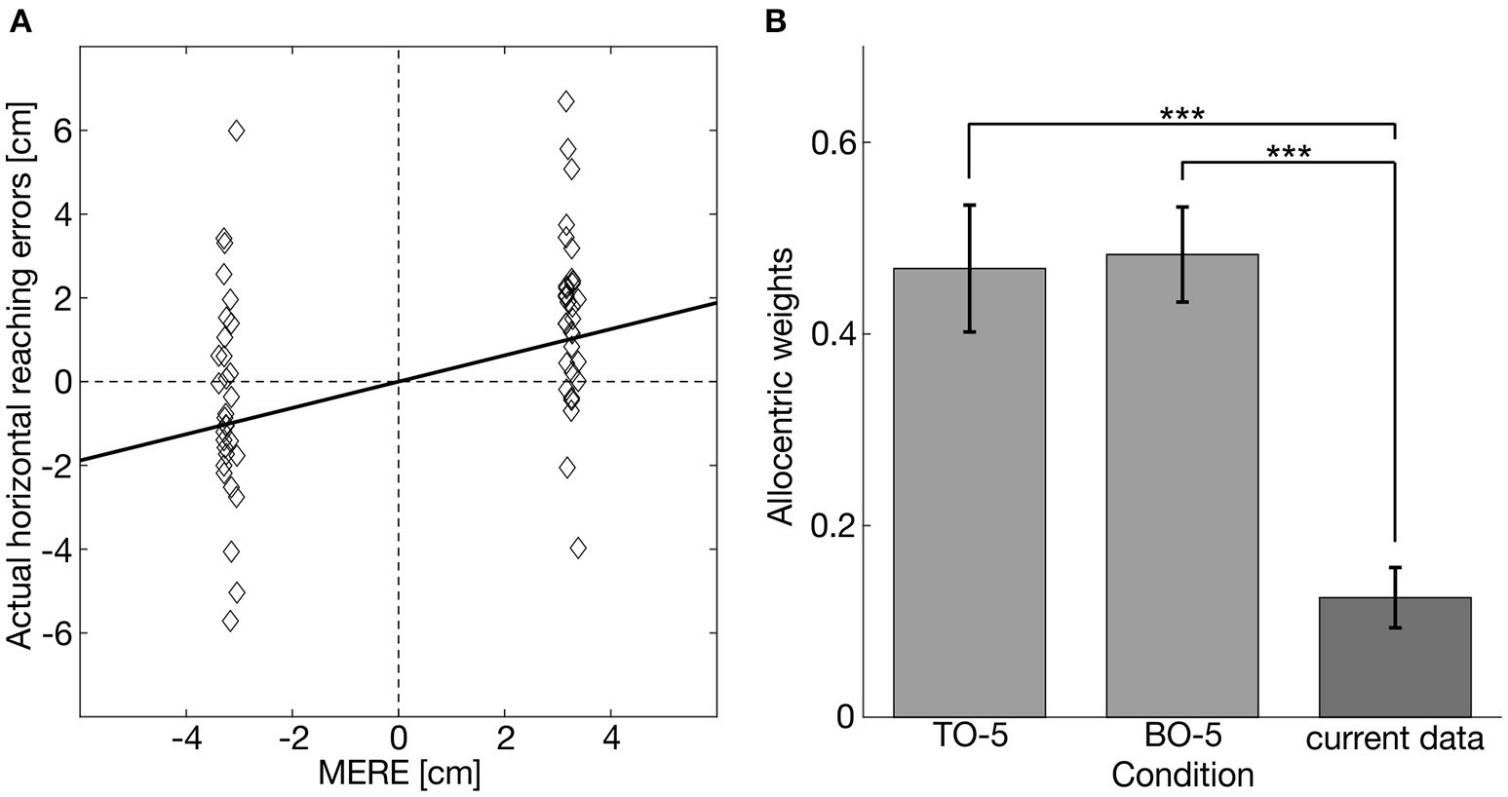
**(A)** Example of a linear fit between MERE and actual horizontal reaching errors for one example participant for the condition RO same. The slope of the fit defines the allocentric weight for this participant in this condition. **(B)** Mean allocentric weights from our previous study (Klinghammer et al., 2015; lighter gray bars) where five table objects (TO-5) or five background objects (BO-5) were shifted in the same direction and our current experiment in which also 5 objects distributed across the table and the background were shifted. Asterisks indicate significant differences (^***^p < 0.001).

Figure 6A depicts the actual reaching errors and the corresponding MERE of one prototypical participant for the condition RO same for leftward (negative values) and rightward (positive values) object displacements. The slope of the regression line defined the allocentric weight of the respective condition. Allocentric weights of all participants were distributed normally in every condition (K-S test: *ps* > 0.335). On the group level, allocentric weights of RO same and RO+IO same significantly differed from zero [RO same: *t*_(16)_ = 3.885, *p* = 0.006; RO+IO same: *t*_(16)_, *p* < 0.001], whereas allocentric weights of the other conditions did not (Table 3).

**Table 3:** **Summary of allocentric weights per condition**.

**Condition**	**Range**	**Mean**	***SD***	***t*-Test results**
RO same	−0.08–0.46	0.13	0.13	*t*_(16)_ = 3.968, *p* = 0.006^**^
RO diff	−0.16–0.13	0.01	0.10	*t*_(16)_ = 0.608, *p* = 0.552
IO same	−0.15–0.29	0.04	0.10	*t*_(16)_ = 1.453, *p* = 0.331
IO diff	−0.10–0.18	0.05	0.09	*t*_(16)_ = 2.075, *p* = 0.218
RO+IO same	−0.00–0.56	0.20	0.14	*t*_(16)_ = 6.017, *p* < 0.001^***^
RO+IO diff	−0.16–0.17	0.04	0.09	*t*_(16)_ = 1.836, *p* = 0.255

Range, mean and standard deviation of the sample are listed. Results of two-sided one-sample t-tests are Bonferroni-Holm corrected. Significant results are indicated by asterisks (

** p < 0.01;

*** *p < 0.001)*.

In order to examine the role of the scene configuration on allocentric coding of reach targets, we compared the allocentric weights of the condition RO same with the corresponding conditions of the previously published experiments (Klinghammer et al., 2015), in which we also shifted five task-relevant objects. We found smaller allocentric weights for the current experiment in which task-relevant and task-irrelevant objects were distributed across the entire scene compared to the previous experiments in which task-relevant and task-irrelevant objects were clustered [current vs. previous - shift of 5 table objects (TO-5): *t*_(15.968)_ = −4.687, *p* < 0.001, *g* = 1.88; current vs. previous–shift of 5 background objects (BO-5): *t*_(24)_ = −6.371; *p* < 0.001, *g* = 2.54; Figure 6B].

**Figure 7:**
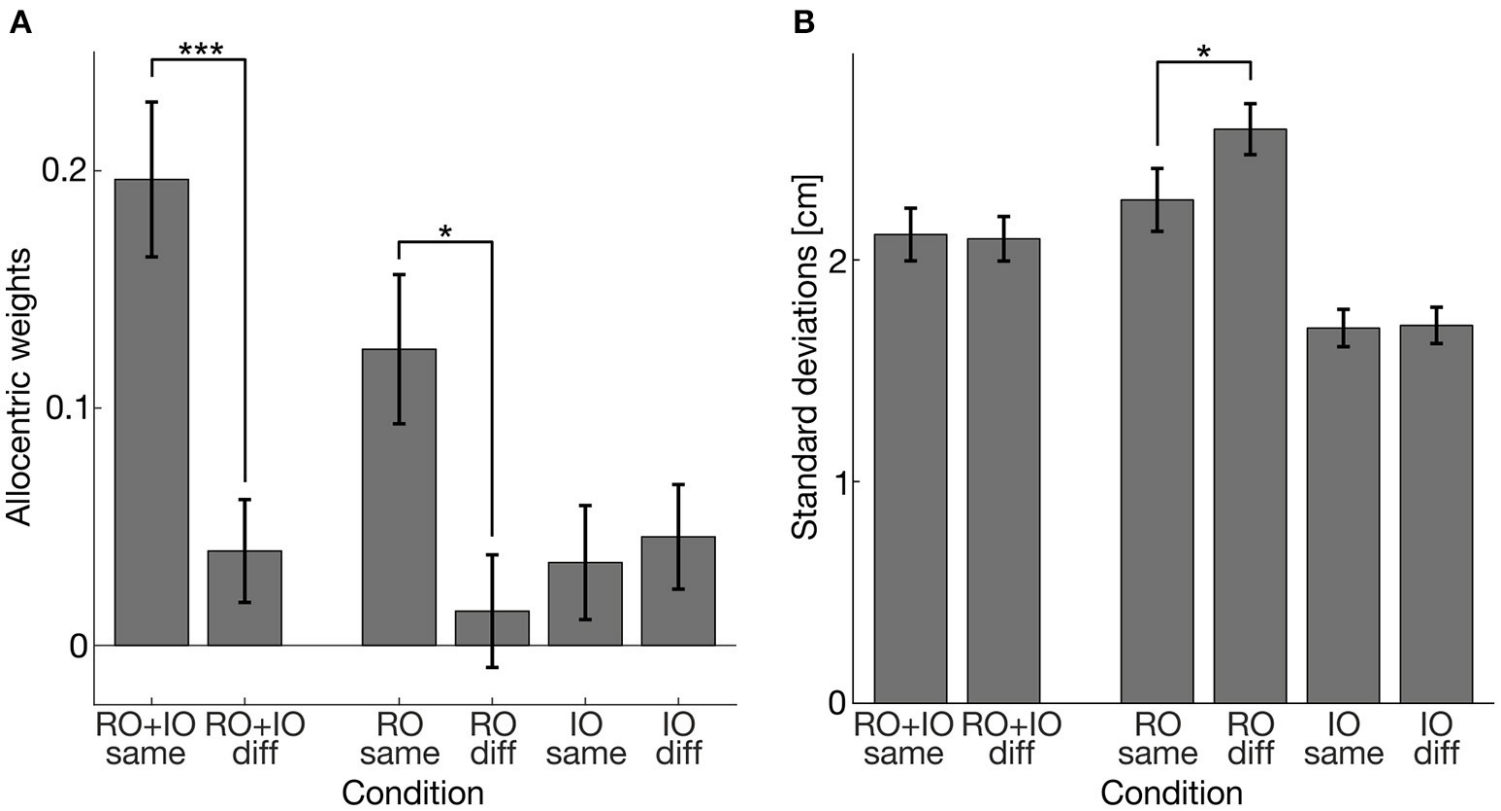
**(A)** Allocentric weights averaged over participants. **(B)** Standard deviations of reaching errors in cm averaged over participants. Error bars indicate 1 *SEM*. Asterisks indicate significant differences (^*^p < 0.05; ^***^p < 0.001).

To assess the influence of the reliability of task-relevant and task-irrelevant objects on their use for allocentric coding, we compared conditions with coherent and incoherent shifts of task-relevant and/or task-irrelevant objects. The results of the allocentric weights and the standard deviations of the reaching endpoints are illustrated in Figure 7. The K-S test also revealed normally distributed standard deviations of reaching errors (*ps* > 0.446). First, we performed a two-sided one-sample *t*-test for the allocentric weights and standard deviations of the conditions RO+IO same and RO+IO diff in which we shifted both task-relevant and task-irrelevant objects. We found that allocentric weights differed between the conditions [*t*_(16)_ = 3.964, *p* = 0.001, *g* = 0.94] showing higher allocentric weights when task-relevant and task-irrelevant objects were shifted coherently in the same direction than shifted incoherently in opposite directions. The standard deviations did not differ between RO+IO same and RO+IO diff [*t*_(16)_ = 0.256, *p* = 0.801]. Additionally, we compared allocentric weights of RO same and RO+IO same and found that allocentric weights were higher when task-relevant and task-irrelevant objects were shifted together in the same direction than when task-relevant objects were shifted alone [*t*_(16)_ = −3.101, *p* = 0.014, *g* = 0.73]. Second, we conducted a two-way repeated measures ANOVA for the conditions RO same, RO diff, IO same, and IO diff. We found a main effect of shift coherence [*F*_(1, 16)_ = 7.399, *p* = 0.015; *eta*^2^ = 0.06]. Coherent object shifts led to higher allocentric weights (*mean* = 0.080) than incoherent shifts (*mean* = 0.030). We also observed an interaction of shift coherence and object relevance [*F*_(1, 16)_ = 5.619, *p* = 0.030, *eta*^2^ = 0.08]. *Post-hoc t*-tests indicated that allocentric weights of RO same were higher than of RO diff [*t*_(16)_ = 2.903, *p* = 0.021, *g* = 0.69], but did not differ between IO same and IO diff [*t*_(16)_ = −0.470, *p* = 0.645]. Two-way repeated measures ANOVA for standard deviations of reaching endpoints revealed main effects of shift coherence [*F*_(1, 16)_ = 1.086, *p* = 0.019, *mean* coherent shifts = 1.982, *mean* incoherent shifts = 2.147] and object relevance [*F*_(1, 16)_ = 86.097, *p* < 0.001, *mean* relevant object shifts = 2.430, *mean* irrelevant object shifts = 1.699]. These main effects were further restricted by an interaction of shift coherence and object relevance [*F*_(1, 16)_ = 6.977, *p* = 0.018]. *Post-hoc t*-tests indicated that standard deviations of reach endpoints in the condition RO same were smaller than in the condition RO diff [*t*_(16)_ = −2.880, *p* = 0.022], while they were comparable for the conditions IO same and IO diff [*t*_(16)_ = −0.243, *p* = 0.811]. Table 4 summarizes the corresponding descriptive statistics.

**Table 4:** **Summary of standard deviations in cm**.

**Condition**	**Range**	**Mean**	***SD***
RO same	1.18–3.42	2.27	0.59
RO diff	1.22–2.63	1.69	0.35
IO same	1.43–3.54	2.12	0.49
IO diff	1.46–3.11	2.10	0.41
RO+IO same	1.85–3.54	2.59	0.47
RO+IO diff	1.26–2.74	1.71	0.34

*Range, mean and standard deviation of the sample are listed*.

## Discussion

In this study, we investigated the use of allocentric information for memory-guided reaching by using naturalistic, complex scenes which are closer to real-life situations than simple laboratory task using abstract stimuli. In particular, we aimed to investigate how the scene configuration influences allocentric coding of target locations for memory-guided reaching. Second, we wanted to know whether and how the reliability of allocentric information, in particular the reliability of task-relevant and task-irrelevant objects, affects their use as allocentric cues. We found that reaching errors systematically deviated in the direction of object shifts, but only when the objects were task-relevant. However, this effect was substantially reduced when task-relevant objects were distributed across the scene compared to a clustered configuration used in our previous study (Klinghammer et al., 2015), and vanished completely when their reliability was low, i.e., they were shifted incoherently. No deviations of reach endpoints were observed in conditions with shifts of only task-irrelevant objects. Moreover, when solely task-relevant objects were shifted incoherently, the variability of reaching endpoints increased compared to coherent shifts of task-relevant objects.

In order to check whether participants' encoding strategies differed depending on the scene configuration, we created fixation density maps with mean fixation densities for every object arrangement during the encoding phase and compared it to the ones in our previous studies (Fiehler et al., 2014; Klinghammer et al., 2015). Here, we observed that task-relevant objects were fixated more frequently than task-irrelevant objects. This supports our previous findings (Fiehler et al., 2014; Klinghammer et al., 2015) showing that overt visual attention is mainly distributed across areas containing task-relevant information. Thus, participants used the same encoding strategy irrespective of whether the task-relevant objects were clustered or distributed across the scene. Our results are in line with previous work on overt visual attention showing that participants' fixation behavior is highly task-dependent with more fixations on task-relevant objects (Land and Hayhoe, 2001; Ballard and Hayhoe, 2009; DeAngelus and Pelz, 2009).

Overall, we found allocentric weights different from zero (= no-shift control condition) in the RO same and RO+IO same conditions suggesting that allocentric information of task-relevant objects was used to encode target locations for memory-guided reaching. Allocentric weights in these conditions ranged from 0.13 to 0.20 (see Table 3) which indicates that reaching endpoints were affected by up to 20% by the shifted task-relevant objects. The remaining percentage could be attributed to the influence of egocentric or additional allocentric reference frames as the environment also provided other, more stable landmarks, e.g., edge of the screen, edge of the black cardboard, or the physical table. In comparison to our previous experiments (Fiehler et al., 2014; Klinghammer et al., 2015), allocentric weights were relatively small, which was confirmed by the statistical comparison of the RO same condition of the current experiment to the corresponding conditions of our previous experiment (Klinghammer et al., 2015), in which we also coherently shifted five task-relevant objects. A main difference between these experiments is the spatial configuration of task-relevant and task-irrelevant objects which were distributed in the current and grouped in the previous experiments. On the first view, the lower allocentric weights for the distributed object configuration seem to confirm other findings showing that participants preferably rely on landmarks in the direct vicinity of the target (Spetch, 1995; Diedrichsen et al., 2004). By placing task-irrelevant objects as close to the target as task-relevant ones, the former may have served as additional but misleading allocentric cues. However, we found that task-relevant but not task-irrelevant objects differed from zero suggesting that task-relevancy but not landmark vicinity determined to which extent objects were used as allocentric cues. This is also supported by the lower reaching endpoint variability in the condition IO same (*mean* = 1.693) than RO same (*mean* = 2.271). Moreover, the fixation behavior during the encoding phase indicates that participants actually ignored task-irrelevant objects and mainly focused on task-relevant ones. In contrast to clustering the objects on the table or in the background, the distributed object configuration we applied here also increased the distance between the target and the task-relevant objects that may have decreased their use as allocentric cue. This is in line with previous findings which show that the influence of allocentric information decreases with an increasing distance between target and landmarks (Krigolson et al., 2007; Camors et al., 2015). However, we cannot exclude an impact of an overall lower reliability of the allocentric information in the current experiment that arises from the trial randomization as discussed earlier.

By introducing conditions in which we shifted objects coherently in the same direction or incoherently in opposite directions, we were aiming to gain insights on how the reliability of allocentric information affects its use for encoding target locations for memory-guided reaching. As expected, we found the highest allocentric weights in the condition in which the coherence of the object relations was maintained between the encoding and the test scene (RO+IO same). Surprisingly, by shifting task-irrelevant objects in the opposite direction of task-relevant ones (RO+IO diff), the allocentric weights were dramatically reduced and did not differ from zero. Even though these objects were task-irrelevant and thus, could be ignored, they had a clear impact on the use of task-relevant objects as allocentric cues. It is likely that by breaking up the coherence of the whole object arrangement, the reliability of the allocentric information was reduced and as a consequence, participants rather relied on egocentric or other, more stable allocentric information. Alternatively, this pattern could be explained by shifts of task-relevant and task-irrelevant objects in opposite directions canceling each other out instead of affecting the overall reliability of allocentric information. If this was true, we would expect similar effects on reach endpoints if we coherently shifted solely task-relevant and solely task-irrelevant objects, because they should have a similar impact on reaching endpoints and thus cancel each other out. However, this was clearly not the case. We observed no influence on reaching endpoints when we solely shifted task-irrelevant objects, but found a clear influence when we solely shifted task-relevant objects. Therefore, we believe that the observed pattern in the RO+IO diff condition cannot be explained by cancelation effects of task-relevant and task-irrelevant object shifts; rather results are consistent with a decrease of the overall reliability of allocentric information. We found a similar pattern of results when we shifted only task-relevant objects in the same or in different directions (RO same, RO diff). As predicted, allocentric weights were higher when the coherence within the group of task-relevant objects was kept intact compared to the condition in which it was broken with allocentric weights being not different from zero. Again, this finding supports a stronger use of allocentric information when its reliability is high. In contrast to the findings by Byrne and Crawford (2010), we found a higher variability of reaching endpoints for low compared to highly coherent object configurations. This further strengthens an important role of the reliability of task-relevant allocentric information for encoding target locations for memory-guided reaching movements. Interestingly, we observed increased allocentric weights for RO+IO same compared to RO same. Even though, a coherent shift of irrelevant objects alone had no influence on reaching endpoints (i.e., no difference of IO same from zero), they may have increased the overall object reliability when they were shifted coherently with task-relevant objects. All in all, it can be concluded that if the reliability of only task-relevant objects is decreased, their use as allocentric cues is substantially reduced. Even though, task-irrelevant objects are not directly used as allocentric cues, they seem to increase the overall contribution of allocentric information by increasing the objects'reliability when shifted coherently with task-relevant objects.

Our results could be explained within the framework of causal Bayesian integration (Körding et al., 2007; Sato et al., 2007) in which two different target modalities are combined in a Bayes-optimal way. This framework further assumes that the weightings of the target modalities are modulated by the probability of both sharing one source or not. Transferred to our paradigm it is reasonable that by shifting objects in an incoherent manner, the causal link between the target location and positions of the other objects (in terms of their incoherent spatial relation) is broken. In that case, causal Bayesian integration discounts the allocentric information by the remaining non-target objects leading to a low weighting of the allocentric information. Moreover, in incoherent shift conditions the variability associated with the allocentric information increases, which in turn further decreases its weighting. In contrast, if objects are shifted in a coherent way, the causal link between the location of the target and the other objects is maintained leading to a lower variability and thus, higher weighting of the allocentric information.

Overall, our findings demonstrate that the use of allocentric information for memory-guided reaching depends on task-relevancy and the scene configuration, in particular the average distance of the reach target to task-relevant objects. If task-relevancy is given, the reliability of allocentric information determines to which extent allocentric cues are used to encode the location of memorized reach targets. Less reliable allocentric cues contribute to a lesser extent and lead to an increased variability of memory-guided reaching movements. However, the reliability of task-relevant allocentric information seems to be further increased by task-irrelevant allocentric cues if they act coherently. Finally, our results demonstrate that by using more naturalistic, complex stimuli containing a variety of environmental information, new insights into the use and nonuse of allocentric information for encoding target locations for memory-guided reaching movements can be found.

## Ethics statement

Local ethical committee of the Justus-Liebig-University Giessen approved the ethic form #2015-00200, October 28 2015. Each participant signed the ethic form before the start of the experiment.

## Author contributions

MK designed experiment, collected and analyzed data, wrote manuscript. GB designed experiment, wrote manuscript. KF designed experiment, wrote manuscript.

### Conflict of interest statement

The authors declare that the research was conducted in the absence of any commercial or financial relationships that could be construed as a potential conflict of interest.

## Footnotes

^1^*World Medical Association Declaration of Helsinki: Ethical Principles for Medical Research Involving Human Subjects*. Available online at: https://www.wma.net/policies-post/wma-declaration-of-helsinki-ethical-principles-for-medical-research-involving-human-subjects/.
